# Interplay between host and *Candida albicans* during commensal gut colonization

**DOI:** 10.1371/journal.ppat.1011607

**Published:** 2023-09-14

**Authors:** Andrew W. Day, Carol A. Kumamoto

**Affiliations:** 1 Graduate School of Biomedical Sciences, Tufts University, Boston, Massachusetts, United States of America; 2 Department of Molecular Biology and Microbiology, Tufts University, Boston, Massachusetts, United States of America; University of Maryland, Baltimore, UNITED STATES

## Introduction

Mammals are naturally colonized with a diverse consortium of microbes composed of bacteria, fungi and other organisms. Although fungi make up a small percentage of the total microbial community, they have significant effects on the gastrointestinal ecosystem. For example, the presence of fungal species in the gut community affects the host immune system [1]. The bacterial community is affected by the presence of fungi and the response of the bacterial community to antibiotic stress is altered when a fungus is introduced. Additionally, fungi affect gut metabolite levels with impacts on the physiology of the host. In this communication, we will discuss some of the ways that the fungus *Candida albicans* interacts with its host, including the beneficial effects of *C*. *albicans* against disease due to *Clostridioides difficile* infection. We also consider the effects of *C*. *albicans* colonization on the gut–brain axis.

### Gut colonizing fungal populations are the sources of organisms that cause invasive fungal infection

Many fungal pathogens such as the primary pathogens *Coccidioides immitis*, *Histoplasma capsulatum*, and *Blastomyces dermatitidis* and the opportunistic pathogens *Cryptococcus neoformans* or *Aspergillus fumigatus* are environmental. These organisms often initiate infection in the lungs after they are inhaled by the host.

On the contrary, *C*. *albicans* does not have a major environmental reservoir and can be acquired by human infants early in life [2], probably from caregivers. *C*. *albicans* is thought to colonize its host as a normal member of the human gut microbiome for long periods of time without causing disease. If the host becomes immunocompromised, colonizing *C*. *albicans* can disseminate from the gut and cause potentially life-threatening candidiasis [3]. Invasive candidiasis is usually caused by endogenous organisms [4].

### Gene expression in colonizing *C*. *albicans* is regulated so that fungal behavior responds to and influences host immune status

During experimental murine colonization, gene expression in *C*. *albicans* cells was found to differ depending on the immune status of the host [5]. Expression of *EFG1*, encoding a key transcription factor that regulates many important activities of *C*. *albicans*, was higher during colonization of healthy mice (BALB/c) and lower during colonization of immunodeficient mice (*nu/nu* BALB/c). Under experimental conditions, immunodeficient mice showed no evidence of disease but the colonizing fungi responded to their immunodeficiency by expressing lower levels of *EFG1*.

The mechanism for coupling gene expression in colonizing fungi to the status of the host’s immune response rests on natural variability in *EFG1* expression from cell to cell and the properties of low Efg1 activity cells [5,6]. These cells can be considered “testers” of the host’s immune status. Changes in *EFG1* expression probably occur early during the transition of a host from an immunocompetent to immunosuppressed state and may herald the change in the fungal population from benign commensals to invasive pathogens.

Variation in expression of another *C*. *albicans* transcription factor, Ume6, during commensal colonization promotes the protective Th17 response of the host [7] providing additional evidence that variability in *C*. *albicans* gene expression impacts the interaction between *C*. *albicans* and the host. These examples illustrate the nuanced interactions between host and *C*. *albicans* that allow each to respond to changes in the other.

### *C*. *albicans* gut colonization alters susceptibility to bacterial infection

Since the status of the host affects *C*. *albicans* gene expression and variation in gene expression alters interaction with the host, it seemed possible that *C*. *albicans* colonization could have far-reaching effects on host physiology. Here, we will discuss effects of colonization on host susceptibility to gastrointestinal infection and on the gut–brain axis.

Several studies show that *C*. *albicans* and bacteria affect each other’s virulence. For example, we analyzed the effect of pre-colonization with *C*. *albicans* on the susceptibility of mice to *Clostridioides* (formerly *Clostridium*) *difficile* infection and demonstrated a protective effect of pre-colonization against this important infection. *C*. *difficile* infection (CDI) is the most common nosocomial infection in the United States and is responsible for considerable morbidity and mortality. We asked whether the presence of *C*. *albicans* in the gut community would alter the course of CDI. Results showed that the presence of *C*. *albicans* was protective against lethal CDI in mice [8].

*C*. *albicans* pre-colonization was associated with changes in the host GI tract. Stronger expression of the protective cytokine IL-17A was detected in *C*. *albicans* pre-colonized mice [8]. Additionally, changes in the gut metabolome associated with *C*. *albicans* colonization affected disease severity [9]. Specifically, elevated levels of unsaturated fatty acids were observed in *C*. *albicans* colonized mice and feeding mice without *C*. *albicans* a source of unsaturated fatty acids (olive oil) was protective against lethal CDI. Further, *C*. *difficile* cultured in laboratory media containing oleic acid (the major fatty acid constituent of olive oil) grew but with reduced expression of toxin genes. Also, *C*. *difficile* cells recovered from the cecal epithelium of olive oil-fed mice exhibited reduced toxin gene expression. The results thus show that *C*. *albicans* colonization changed the gut metabolite environment in a way that reduced *C*. *difficile* virulence.

*C*. *albicans* is not the only organism that is protective against *C*. *difficile* infection. Commensal bacteria such as *Clostridium scindens* [10], Lachnospiraceae [11], and *Paraclostridium bifermentans* [12] are also protective against murine CDI. The mechanisms involved in protection differ, but these results show that multiple commensal organisms can protect against this bacterial infection.

*C*. *albicans* shows interaction with other bacterial species. Pre-colonization with *C*. *albicans* reduced the virulence of *Pseudomonas aeruginosa* following oral inoculation of neutropenic mice [13]. In contrast, some combinations of *C*. *albicans* and bacteria have the opposite effect and increase virulence [14–16]. Additionally, some bacteria such as *Enterococcus* and *Pseudomonas* express antifungal activities [17,18]. These findings demonstrate that *C*. *albicans–*bacterial interactions affect virulence in multiple ways and are consequential for the host.

### *C*. *albicans* colonization affects the gut–brain axis

Impacts of the gut microbiota on the brain through the gut–brain axis have recently become the subject of investigation. The gut–brain axis is defined as the bidirectional communication between the brain and the gastrointestinal tract through direct and indirect pathways including direct neuronal innervation of the intestines, metabolic pathways, and immune system modulation. All of these pathways can be impacted by gastrointestinal microbes or microbial products and can impact activities in the brain.

Gastrointestinal colonization by *C*. *albicans* resulted in altered behavior in mice [19]. Mice colonized with *C*. *albicans* had elevated afternoon corticosterone and an anxiety-like phenotype due to dysregulation of lipid metabolism and perturbation of the endocannabinoid system [19]. Endocannabinoids are signaling lipids that regulate synaptic transmission and modulate inflammation, neurological development, memory formation, anxiety, and depression [20]. Based on the functions of the endocannabinoid system, it is likely that *C*. *albicans* or other microbes that alter the endocannabinoid system would have other behavioral impacts. In fact, in a chronic unpredictable stress model, microbiota dysbiosis and endocannabinoid perturbations contributed to the development of depressive-like behavior [21].

Furthermore, *C*. *albicans* affects other pathways such as immune responses that could modulate neuroinflammation. Additionally, *C*. *albicans* impacts plasma levels of ketones such as β-hydroxybutyrate [22], a lipid molecule that has neuroprotective effects. β-hydroxybutyrate was reduced in mice colonized with microbiomes from humans with alcohol use disorder [23] and this reduction correlated with depression-like behavior and altered social behavior in these mice.

In summary, the gut–brain axis is extremely complex. Many different microorganisms are involved in its modulation through a number of pathways, leading to a variety of microbiota-responsive neuropsychological phenotypes.

### Possible contributions of *C*. *albicans* and gut microbiota to the evolutionary fitness of the host

Why does *C*. *albicans* have such profound effects on its host? As discussed above, *C*. *albicans* is not the only organism to affect susceptibility to bacterial infection or signaling in the gut–brain axis. Other members of the gut microbiota have similar effects. These observations suggest that the impacts of microbiota on the host could represent an important way to generate diversity in a human population. Microbiota diversity could protect the population as a whole from extinction due to endemic or invasive pathogens that would otherwise cause widespread mortality in a genetically similar population. Additionally, variability in microbiota composition between humans could have very individual effects on each person’s gut–brain axis. As a result, each person’s response to a stressful condition could be slightly different. Diversity in responses to danger may promote survival of a group by increasing the chances that the group will find an optimal response. In small family groups where genetic diversity may not be high, microbiota diversity may provide a needed mechanism that allows the group to sample a wider variety of responses to danger. The microbiota–gut–brain axis thus provides survival benefits in a cruel world.

## References

[1] d’Enfert C, Kaune AK, Alaban LR, Chakraborty S, Cole N, Delavy M, et al. The impact of the Fungus-Host-Microbiota interplay upon *Candida albicans* infections: current knowledge and new perspectives. FEMS Microbiol Rev. 2021;45(3). Epub 2020/11/25. doi: 10.1093/femsre/fuaa060 ; PubMed Central PMCID: PMC8100220. 33232448 PMC8100220

[2] Russell C, Lay KM. Natural history of *Candida* species and yeasts in the oral cavities of infants. Arch Oral Biol. 1973;18(8):957–62. Epub 1973/08/01. doi: 10.1016/0003-9969(73)90176-3 . 4581575

[3] Sprague JL, Kasper L, Hube B. From intestinal colonization to systemic infections: *Candida albicans* translocation and dissemination. Gut Microbes. 2022;14(1):2154548. Epub 2022/12/13. doi: 10.1080/19490976.2022.2154548 ; PubMed Central PMCID: PMC9746630. 36503341 PMC9746630

[4] Zhai B, Ola M, Rolling T, Tosini NL, Joshowitz S, Littmann ER, et al. High-resolution mycobiota analysis reveals dynamic intestinal translocation preceding invasive candidiasis. Nat Med. 2020;26(1):59–64. Epub 2020/01/08. doi: 10.1038/s41591-019-0709-7 ; PubMed Central PMCID: PMC7005909. 31907459 PMC7005909

[5] Pierce JV, Kumamoto CA. Variation in *Candida albicans EFG1* expression enables host-dependent changes in colonizing fungal populations. mBio. 2012;3(4):e00117–12. Epub 2012/07/26. doi: 10.1128/mBio.00117-12 ; PubMed Central PMCID: PMC3413400. 22829676 PMC3413400

[6] Pierce JV, Dignard D, Whiteway M, Kumamoto CA. Normal adaptation of *Candida albicans* to the murine gastrointestinal tract requires Efg1p-dependent regulation of metabolic and host defense genes. Eukaryot Cell. 2013;12(1):37–49. Epub 2012/11/06. doi: 10.1128/EC.00236-12 ; PubMed Central PMCID: PMC3535844. 23125349 PMC3535844

[7] Shao TY, Kakade P, Witchley JN, Frazer C, Murray KL, Ene IV, et al. *Candida albicans* oscillating *UME6* expression during intestinal colonization primes systemic Th17 protective immunity. Cell Rep. 2022;39(7):110837. Epub 2022/05/19. doi: 10.1016/j.celrep.2022.110837 ; PubMed Central PMCID: PMC9196946. 35584674 PMC9196946

[8] Markey L, Shaban L, Green ER, Lemon KP, Mecsas J, Kumamoto CA. Pre-colonization with the commensal fungus *Candida albicans* reduces murine susceptibility to *Clostridium difficile* infection. Gut Microbes. 2018;9(6):497–509. Epub 2018/04/19. doi: 10.1080/19490976.2018.1465158 ; PubMed Central PMCID: PMC6287688. 29667487 PMC6287688

[9] Romo JA, Markey L, Kumamoto CA. Lipid Species in the GI Tract are Increased by the Commensal Fungus *Candida albicans* and Decrease the Virulence of *Clostridioides difficile*. J Fungi (Basel). 2020;6(3). Epub 2020/07/09. doi: 10.3390/jof6030100 ; PubMed Central PMCID: PMC7557729. 32635220 PMC7557729

[10] Buffie CG, Bucci V, Stein RR, McKenney PT, Ling L, Gobourne A, et al. Precision microbiome reconstitution restores bile acid mediated resistance to *Clostridium difficile*. Nature. 2015;517(7533):205–8. Epub 2014/10/23. doi: 10.1038/nature13828 ; PubMed Central PMCID: PMC4354891. 25337874 PMC4354891

[11] Reeves AE, Koenigsknecht MJ, Bergin IL, Young VB. Suppression of *Clostridium difficile* in the gastrointestinal tracts of germfree mice inoculated with a murine isolate from the family Lachnospiraceae. Infect Immun. 2012;80(11):3786–94. Epub 2012/08/15. doi: 10.1128/IAI.00647-12 ; PubMed Central PMCID: PMC3486043. 22890996 PMC3486043

[12] Girinathan BP, DiBenedetto N, Worley JN, Peltier J, Arrieta-Ortiz ML, Immanuel SRC, et al. In vivo commensal control of *Clostridioides difficile* virulence. Cell Host Microbe. 2021;29(11):1693–708 e7. Epub 2021/10/13. doi: 10.1016/j.chom.2021.09.007 ; PubMed Central PMCID: PMC8651146. 34637781 PMC8651146

[13] Lopez-Medina E, Fan D, Coughlin LA, Ho EX, Lamont IL, Reimmann C, et al. *Candida albicans* Inhibits *Pseudomonas aeruginosa* Virulence through Suppression of Pyochelin and Pyoverdine Biosynthesis. PLoS Pathog. 2015;11(8):e1005129. Epub 2015/08/28. doi: 10.1371/journal.ppat.1005129 ; PubMed Central PMCID: PMC4552174. 26313907 PMC4552174

[14] Todd OA, Fidel PL Jr., Harro JM, Hilliard JJ, Tkaczyk C, Sellman BR, et al. *Candida albicans* Augments *Staphylococcus aureus* Virulence by Engaging the Staphylococcal *agr* Quorum Sensing System. mBio. 2019;10(3). Epub 2019/06/06. doi: 10.1128/mBio.00910-19 ; PubMed Central PMCID: PMC6550526. 31164467 PMC6550526

[15] Bergeron AC, Seman BG, Hammond JH, Archambault LS, Hogan DA, Wheeler RT. *Candida albicans* and *Pseudomonas aeruginosa* Interact To Enhance Virulence of Mucosal Infection in Transparent Zebrafish. Infect Immun. 2017;85(11). Epub 2017/08/30. doi: 10.1128/IAI.00475-17 ; PubMed Central PMCID: PMC5649025. 28847848 PMC5649025

[16] Shing SR, Ramos AR, Patras KA, Riestra AM, McCabe S, Nizet V, et al. The Fungal Pathogen *Candida albicans* Promotes Bladder Colonization of Group B *Streptococcus*. Front Cell Infect Microbiol. 2019;9:437. Epub 2020/01/31. doi: 10.3389/fcimb.2019.00437 ; PubMed Central PMCID: PMC6966239. 31998657 PMC6966239

[17] Cruz MR, Cristy S, Guha S, De Cesare GB, Evdokimova E, Sanchez H, et al. Structural and functional analysis of EntV reveals a 12 amino acid fragment protective against fungal infections. Nat Commun. 2022;13(1):6047. Epub 2022/10/14. doi: 10.1038/s41467-022-33613-1 ; PubMed Central PMCID: PMC9562342 peptides described in this work. The other authors declare no competing interests. 36229448 PMC9562342

[18] Morales DK, Jacobs NJ, Rajamani S, Krishnamurthy M, Cubillos-Ruiz JR, Hogan DA. Antifungal mechanisms by which a novel *Pseudomonas aeruginosa* phenazine toxin kills *Candida albicans* in biofilms. Mol Microbiol. 2010;78(6):1379–92. Epub 2010/12/15. doi: 10.1111/j.1365-2958.2010.07414.x ; PubMed Central PMCID: PMC3828654. 21143312 PMC3828654

[19] Markey L, Hooper A, Melon LC, Baglot S, Hill MN, Maguire J, et al. Colonization with the commensal fungus *Candida albicans* perturbs the gut-brain axis through dysregulation of endocannabinoid signaling. Psychoneuroendocrinology. 2020;121:104808. Epub 2020/08/03. doi: 10.1016/j.psyneuen.2020.104808 ; PubMed Central PMCID: PMC7572798. 32739746 PMC7572798

[20] Osafo N, Yeboah OK, Antwi AO. Endocannabinoid system and its modulation of brain, gut, joint and skin inflammation. Mol Biol Rep. 2021;48(4):3665–80. Epub 2021/04/29. doi: 10.1007/s11033-021-06366-1 . 33909195

[21] Chevalier G, Siopi E, Guenin-Mace L, Pascal M, Laval T, Rifflet A, et al. Effect of gut microbiota on depressive-like behaviors in mice is mediated by the endocannabinoid system. Nat Commun. 2020;11(1):6363. Epub 2020/12/15. doi: 10.1038/s41467-020-19931-2 ; PubMed Central PMCID: PMC7732982. 33311466 PMC7732982

[22] Peroumal D, Sahu SR, Kumari P, Utkalaja BG, Acharya N. Commensal Fungus *Candida albicans* Maintains a Long-Term Mutualistic Relationship with the Host To Modulate Gut Microbiota and Metabolism. Microbiol Spectr. 2022;10(5):e0246222. Epub 2022/09/23. doi: 10.1128/spectrum.02462-22 ; PubMed Central PMCID: PMC9603587. 36135388 PMC9603587

[23] Leclercq S, Le Roy T, Furgiuele S, Coste V, Bindels LB, Leyrolle Q, et al. Gut Microbiota-Induced Changes in beta-Hydroxybutyrate Metabolism Are Linked to Altered Sociability and Depression in Alcohol Use Disorder. Cell Rep. 2020;33(2):108238. Epub 2020/10/15. doi: 10.1016/j.celrep.2020.108238 . 33053357

